# A Wearable, Steerable, Transcranial Low‐Intensity Focused Ultrasound System

**DOI:** 10.1002/jum.16600

**Published:** 2024-10-24

**Authors:** Christopher R. Bawiec, Peter J. Hollender, Sarah B. Ornellas, Jessica N. Schachtner, Jacob F. Dahill‐Fuchel, Soren D. Konecky, John J. B. Allen

**Affiliations:** ^1^ Openwater San Francisco California USA; ^2^ Department of Psychology University of Arizona Tucson Arizona USA

**Keywords:** low‐intensity focused ultrasound (LIFU), neuromodulation, therapeutic ultrasound, transcranial focused ultrasound (tFUS)

## Abstract

**Objectives:**

Transcranial low‐intensity focused ultrasound (LIFU) offers unique opportunities for precisely neuromodulating small and/or deep targets within the human brain, which may be useful for treating psychiatric and neurological disorders. This article presents a novel ultrasound system that delivers focused ultrasound through the forehead to anterior brain targets and evaluates its safety and usability in a volunteer study.

**Methods:**

The ultrasound system and workflow are described, including neuronavigation, LIFU planning, and ultrasound delivery components. Its capabilities are analyzed through simulations and experiments in water to establish its safe steering range. A cohort of 20 healthy volunteers received a LIFU protocol aimed at the anterior medial prefrontal cortex (amPFC), using imaging and questionnaires to screen for adverse effects. Additional development after the study also analyzes the effect of the skull and sinus cavities on delivered ultrasound energy.

**Results:**

Simulations and hydrophone readings agreed with <5% error, and the safe steering range was found to encompass a 1.8 cm × 2.5 cm × 2 cm volume centered at a depth 5 cm from the surface of the skin. There were no adverse effects evident on qualitative assessments, nor any signs of damage in susceptibility‐weighted imaging scans. All participants tolerated the treatment well. The interface effectively enabled the users to complete the workflow with all participants. In particular, the amPFC of every participant was within the steering limits of the system. A post hoc analysis showed that “virtual fitting” could aid in steering the beams around subjects' sinuses.

**Conclusions:**

The presented system safely delivered LIFU through the forehead while targeting the amPFC in all volunteers, and was well‐tolerated. With the capabilities validated here and positive results of the study, this technology appears well‐suited to explore LIFU's efficacy in clinical neuromodulation contexts.

**Abbreviations**
AE: Adverse Events
amPFC: Anterial Medial Prefrontal Cortex
ISPTA: Intensity, Spatial‐Peak, Time Average
LIFU: Low Intensity Focused Ultrasound
MI: Mechanical Index
MRI: Magnetic Resonance Imagine
PNP: Peak Negative Pressure
PRI: Pulse Repetition Interval
PTRI: Pulse Train Repetition Interval
tFUS: Transcranial Focused Ultrasound
TIC: Thermal Index of the Cranium
TMM: Tissue Mimicking Material

Transcranial‐focused ultrasound (tFUS) used at low acoustic intensities is an emerging technique for non‐invasive neuromodulation with improved spatial resolution and targeting compared with magnetic or electric non‐invasive brain stimulation.^1^ Several studies have been performed using tFUS for non‐invasive neurostimulation of targeted brain regions, including the primary motor cortex and hippocampus,^2^ amygdala,^3^ and thalamus.^4^ The first human transcranial application of focused ultrasound neuromodulation involved stimulation of the frontal cortex applied on 31 patients affected by chronic pain.^5^ Subsequent use of the tFUS technique was described targeting the primary somatosensory cortex of healthy volunteers, in a within‐patients, sham‐controlled study.^6^ There has been significantly increased interest in the clinical community on the applications of tFUS on neuromodulation, with several recent reviews published to summarize the state of the art on this topic.^1^, ^7^, ^8^, ^9^, ^10^

In particular, a systematic meta‐analyses on tFUS safety across 33 studies performed in both humans and animals has demonstrated a favorable safety profile.^11^ Early results of measured clinical effects of tFUS in modulating mood and functional connectivity by targeting the right inferior frontal gyrus (rIFG), an area implicated in mood and emotional regulation, have shown promising signals.^12^ Briefly, in a randomized, placebo‐controlled, double‐blind study, participants who received tFUS reported an overall increase in Global Affect, an aggregate score from the Visual Analog Mood Scales^13^ indicating a positive shift in mood, which was also objectively indexed via a decrease in functional magnetic resonance imaging (fMRI) resting‐state functional connectivity (FC) within resting‐state networks related to emotion and mood regulation.^12^ These results support that tFUS may be useful as a safe and effective^14^, ^15^ means of noninvasive transcranial brain stimulation that has the potential of modulating mood and emotional regulation networks in the prefrontal cortex. Additionally, tFUS's ability to reach^16^, ^17^, ^18^, ^19^, ^20^ targets deeper than the surface of the brain may open new opportunities for treating more conditions, improving effectiveness.

A review by Javid et al^21^ highlights that most animal or human tFUS studies commonly adopt single‐element, unfocused ultrasound transducers,^2^, ^3^, ^6^ which offer low spatial resolution in the sub‐MHz range usually employed for neuromodulation purposes,^7^ also requiring mechanical move the transducer for every new intended target. To increase the spatial resolution, acoustic lenses can be used, but to achieve electronic steering, a phased array is necessary.^22^ While there is a current phased array system in the market capable of delivering ultrasound energy inside the skull,^23^ the bulky system requires magnetic resonance imaging (MRI) for the entirety of treatment, which is not ideal for recurring treatment schedules. Therefore, there is a pressing need for a wearable, steerable tFUS system that can be used in neuromodulation without requiring hospital infrastructure.

## Methods

### Neuromodulation System

We present the design of a wearable system for delivering transcranial low‐intensity focused ultrasound (LIFU). The system enables the LIFU energy to be delivered through the forehead via a steerable matrix array transducer to anterior targets in the brain. The steerable beam allows one to deliver focused ultrasound energy to targets within a broad region of prefrontal cortex that is implicated in many psychiatric and neurological disorders.

#### Overview

**Figure 1:**
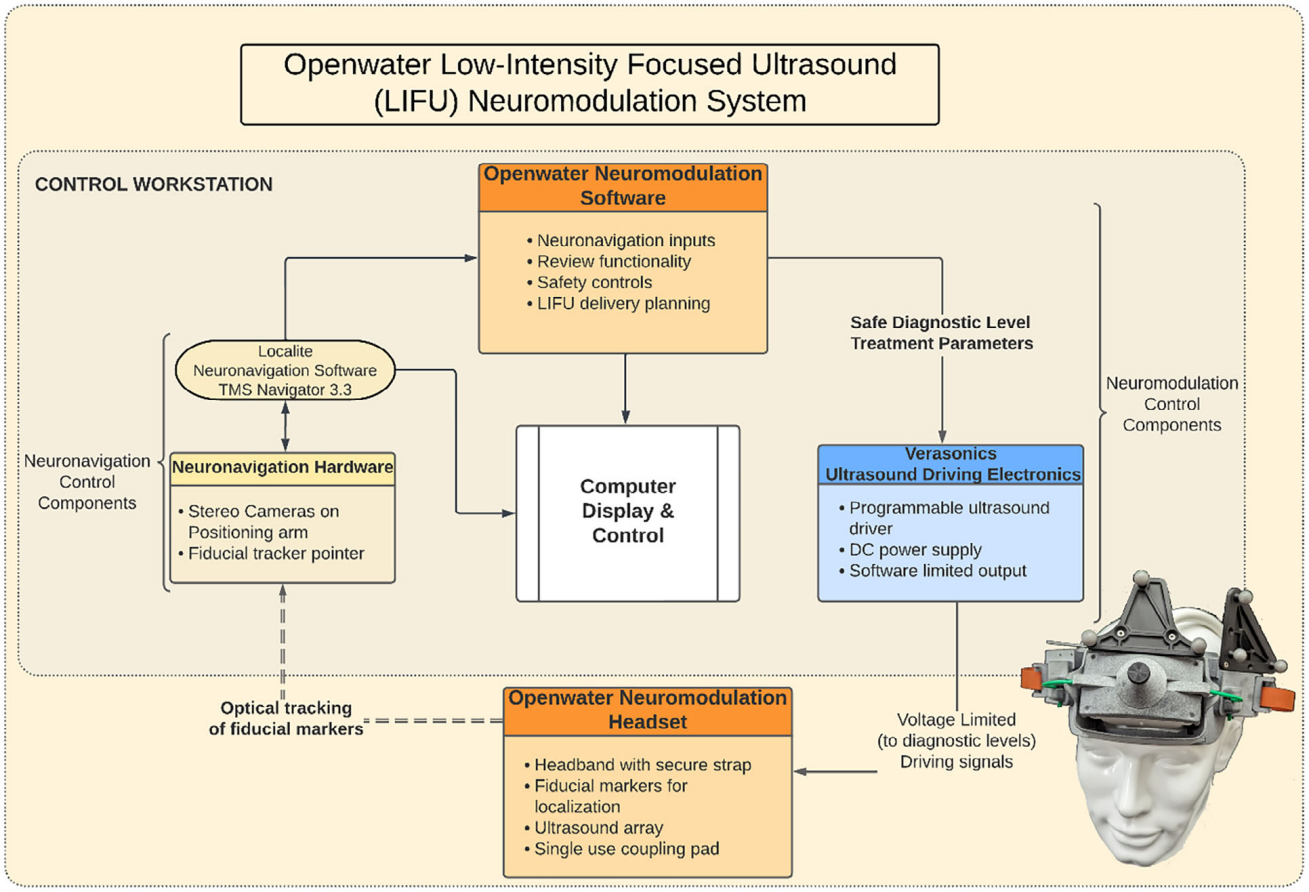
Diagram of the openwater low‐intensity focused ultrasound neuromodulation system components.

**Figure 2:**
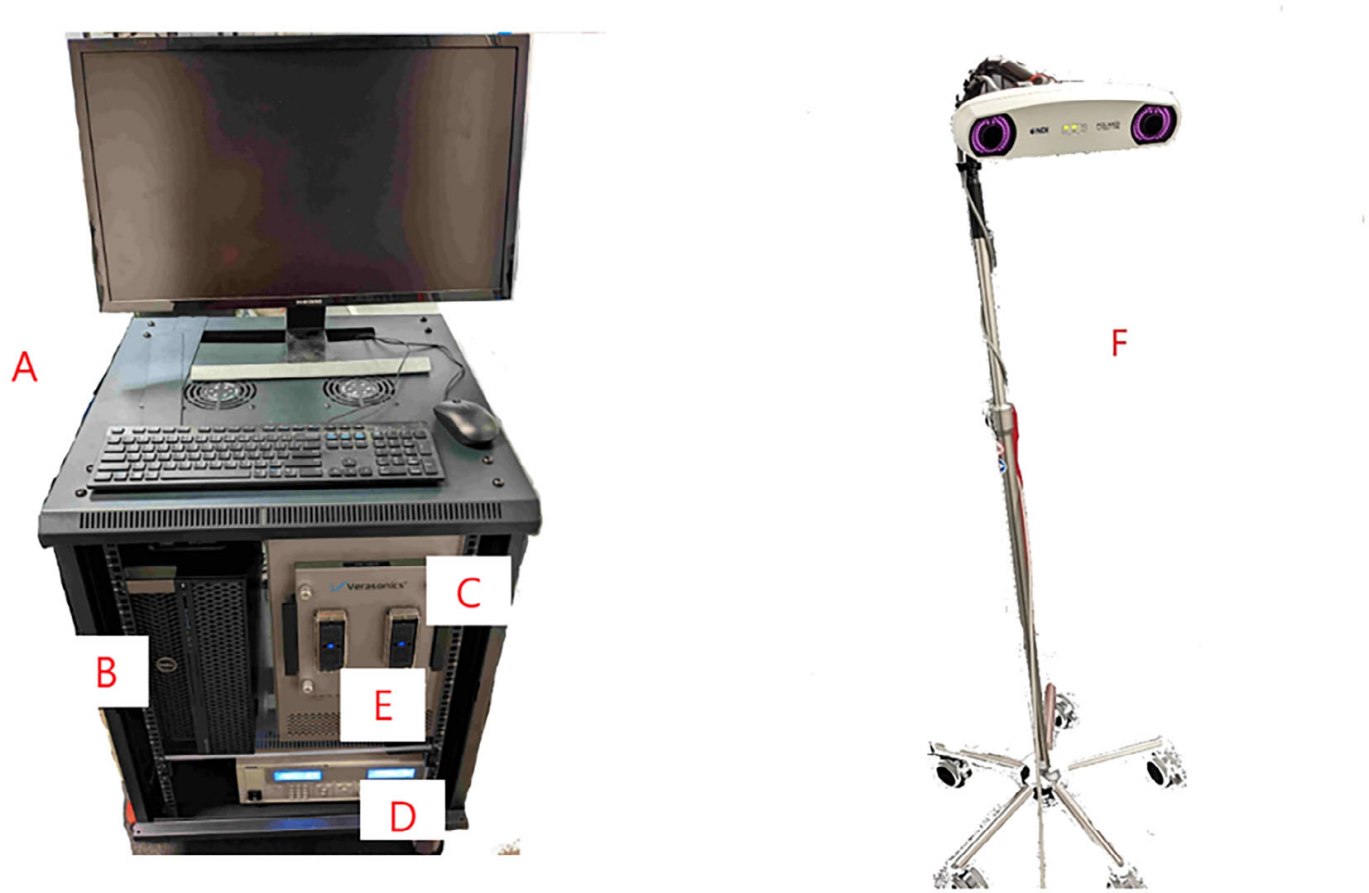
Photo of the system components, (**A**) Cart with monitor keyboard, and mouse, (**B**) Computer, (**C**) Driving electronics, (**D**) DC power supply, (**E**) Connector for ultrasound probe, and (**F**) Rolling pole that holds stereo camera for neuronavigation.

The prototype system has four main components. They are shown in the block diagram in Figure 1. The first component is the neuromodulation headset, a wearable headset containing a custom ultrasound matrix array. The second component is a neuronavigation system for registering the headset and array to the participant's MRI data for coarse targeting of the ultrasound beam through array placement. The third component is the neuromodulation software, which is used to plan LIFU delivery parameters and determine the signals necessary to precisely steer the ultrasound beam to the participant‐specific target location. The fourth and final component is the ultrasound driving electronics, used to apply the electrical signals to the array and deliver the LIFU energy to the target location. Figure 2 shows the driving electronics and stereo camera used for neuronavigation.

#### Headset

##### Ultrasound transducer array

**Figure 3:**
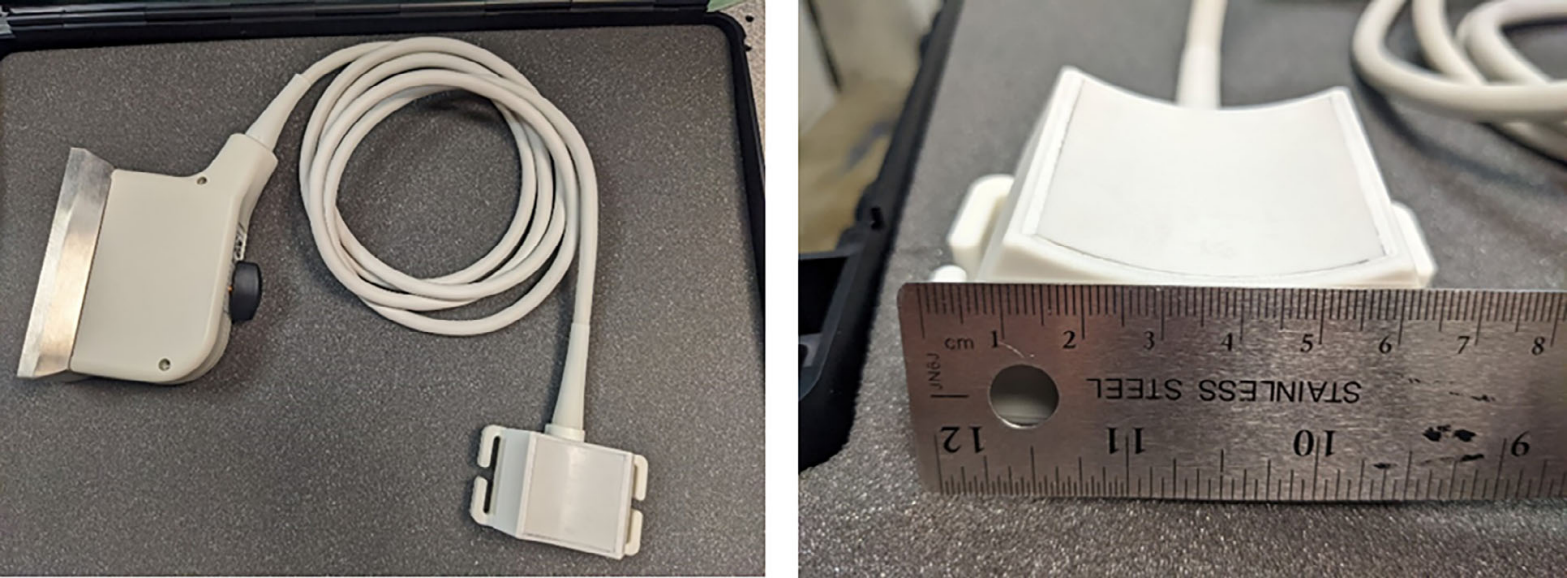
Custom curved‐matrix transcranial ultrasound array.

The neuromodulation ultrasound array consists of a two‐dimensional (2D) array that is designed to be placed and held on the forehead of an individual by a headset superstructure. The custom 400 kHz 2D array consists of 128 ultrasound elements in 11 rows and 12 columns, with the corner elements not connected. The array is cylindrically focused with a radius of curvature of 50 mm in the horizontal direction. There are two thermistors located in the face of the array's corners to allow temperature monitoring during the tFUS delivery. The elements are square and have a 4.1 mm pitch making the surface area ~21.5 cm^2^. The array has a 2‐m insulated cable sleeve that carries all of the control and sensing cables from the array to the ultrasound driving electronics. This flexible connection allows freedom of movement for the participant to position themselves comfortably during the session. The cables are routed to a 260‐pin Cannon ITT Zero Insertion Force connector through custom PCB boards that have electrical matching components (inductors) soldered to them. These inductors are connected in series to cancel out the capacitive impedance of the transducers array elements at 400 kHz. An image of the transducer is shown in Figure 3.

Three retroreflective spheres with a known geometry are affixed to the array to assist with positioning via the neuronavigation sub‐system (discussed in the following section). The three spheres have unique distances and serve as triangulation points for the headset and the array, allowing the neuronavigation subsystem to differentiate and track the instruments with high accuracy. The array has a curvature that allows comfortable placement along the anatomical lines of the participant's forehead.

The ultrasound array's focus is electronically steerable within a steering range, allowing for flexibility in the placement of the transducer without compromising targeting accuracy. The following two criteria were used to define steering range: First, the intended focus spot must be created without generating grating lobes that have peak pressures >50% of the primary focus; second, the acoustic power that is needed to generate a desired focal spot must not exceed that which would cause the Thermal Index of the Cranium (TIC) to be at or above 3. These limitations mitigate the delivery of ultrasound energy to unintended targets in the brain and allow for an ultrasound duration of 10 minutes, respectively.

##### Wearable housing and transducer coupling

**Figure 4:**
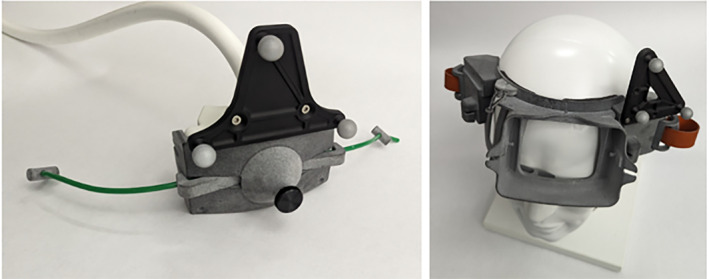
(Left) transducer array in 3D printed housing with fiducial markers. (Right) 3D printed headset with attached fiducial markers on mannequin head, showing opening on the forehead where the transducer array is placed.

The wearable component of the system is a three‐dimensional (3D) printed headset that fits securely on a participant's head and provides an attachment mechanism for the ultrasound transducer array (Figure 4). The design of the headset allows for the one‐dimensional movement of the probe up to 2 cm horizontally along the forehead without repositioning the entire headset. One set of fiducial markers is placed on the headset, which is securely fastened to the participant, for anatomical registration to their MR image. A second set of fiducial markers is placed on the transducer housing and tracks the location of the array when its horizontal position is adjusted. The headset, including the transducer, weights 2.5 lbs.

**Figure 5:**
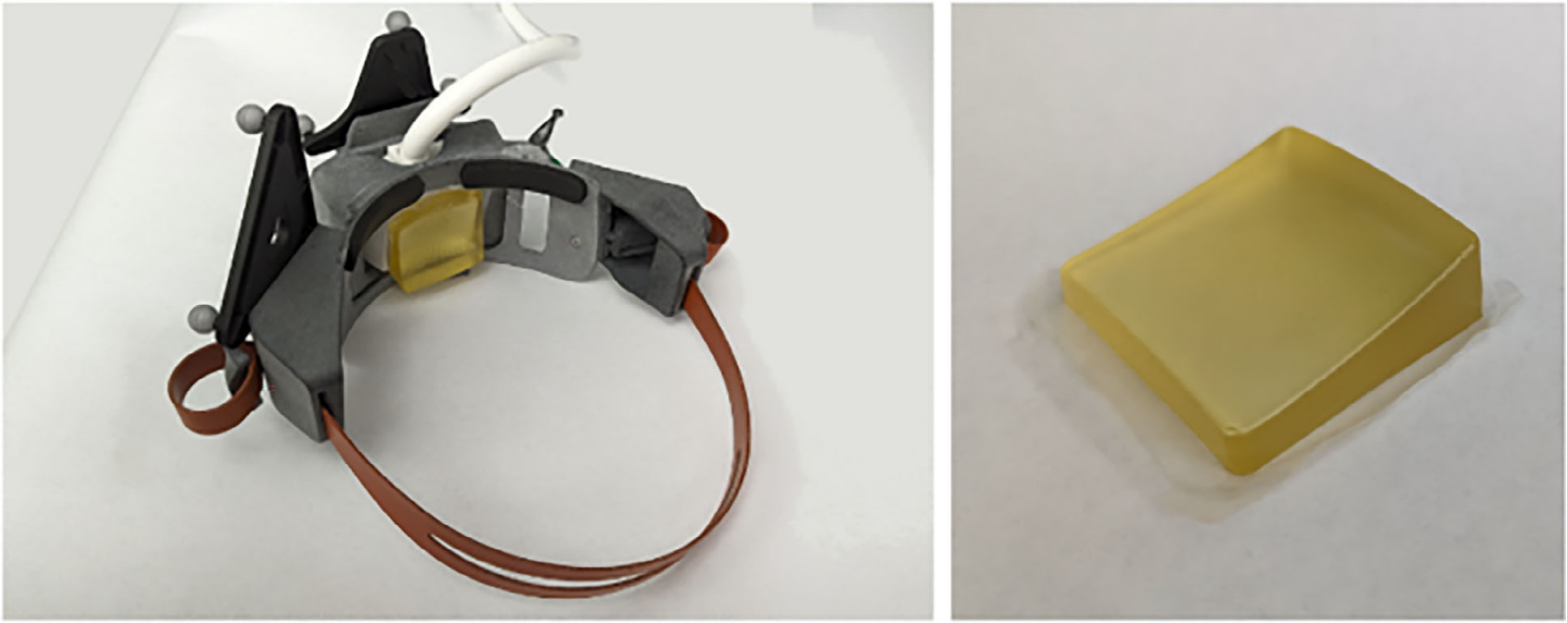
(Left) Headset with transducer in place. (Right) Wedged coupling pad used to couple acoustic energy into the head.

The headset has a central positioning support on the lower portion just above the nose that allows it to be placed along the participant's brow line, between the eyebrows. A custom molded polymer gel pad is placed on the array face and a thin layer of ultrasound gel is placed between the pad and the participant's skin, assuring that the ultrasound can travel through an acoustically equivalent medium until it reaches the target tissue inside the brain. The polymer gel pad is fabricated using a custom designed plano‐convex mold such that when placed on the array the curvature would match a typical forehead. For targeting of the amPFC, the molded polymer gel pad used to acoustically couple the array to the participant was fabricated with a 10° wedge such that it angled the array downwards. The combination of the horizontal movement, the central positioning support, and the wedged coupling pad allowed for positioning of the array within steering bounds of this target (Figure 5).

#### Neuronavigation System

To precisely localize the ultrasound transducer array with respect to each individual's specific anatomy and coregistered medical image, a Localite neuronavigation system (Localite, Bonn, Germany) is used. This neuronavigation system enables the co‐registration of the individual's MR image to their specific anatomy, followed by the registration of the ultrasound transducer array to their anatomy and medical image, using a stereo camera positioned on a pole to detect the position and orientation of objects marked with retroreflective fiducials within its field of view. The accuracy of the camera used by the system is reported by the manufacturer to be 0.35 mm RMS.

Once the patient's MRI is coregistered to their anatomical features and mapped to Montreal Neurological Institute (MNI) coordinates by trained operators following the step‐by‐step procedure in the Localite TNSNavigator software, the anatomical target is loaded and appears in the volume. The transducer, attached to its fiducial tracker, is then coregistered into the neuronavigation system software using a custom calibration technique. The transducer is then positioned over the general target location so that it aims approximately at the target. In contrast to fixed‐focus systems, this alignment only needs to be approximate, because the steerable nature of the matrix array allows the planning software to aim the beam at any target within its steering range.

#### tFUS Delivery Planning and Delivery Software

**Figure 6:**
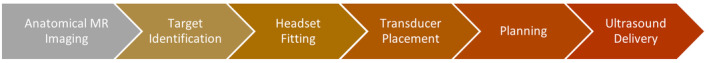
Ultrasound planning and delivery sequence.

**Figure 7:**
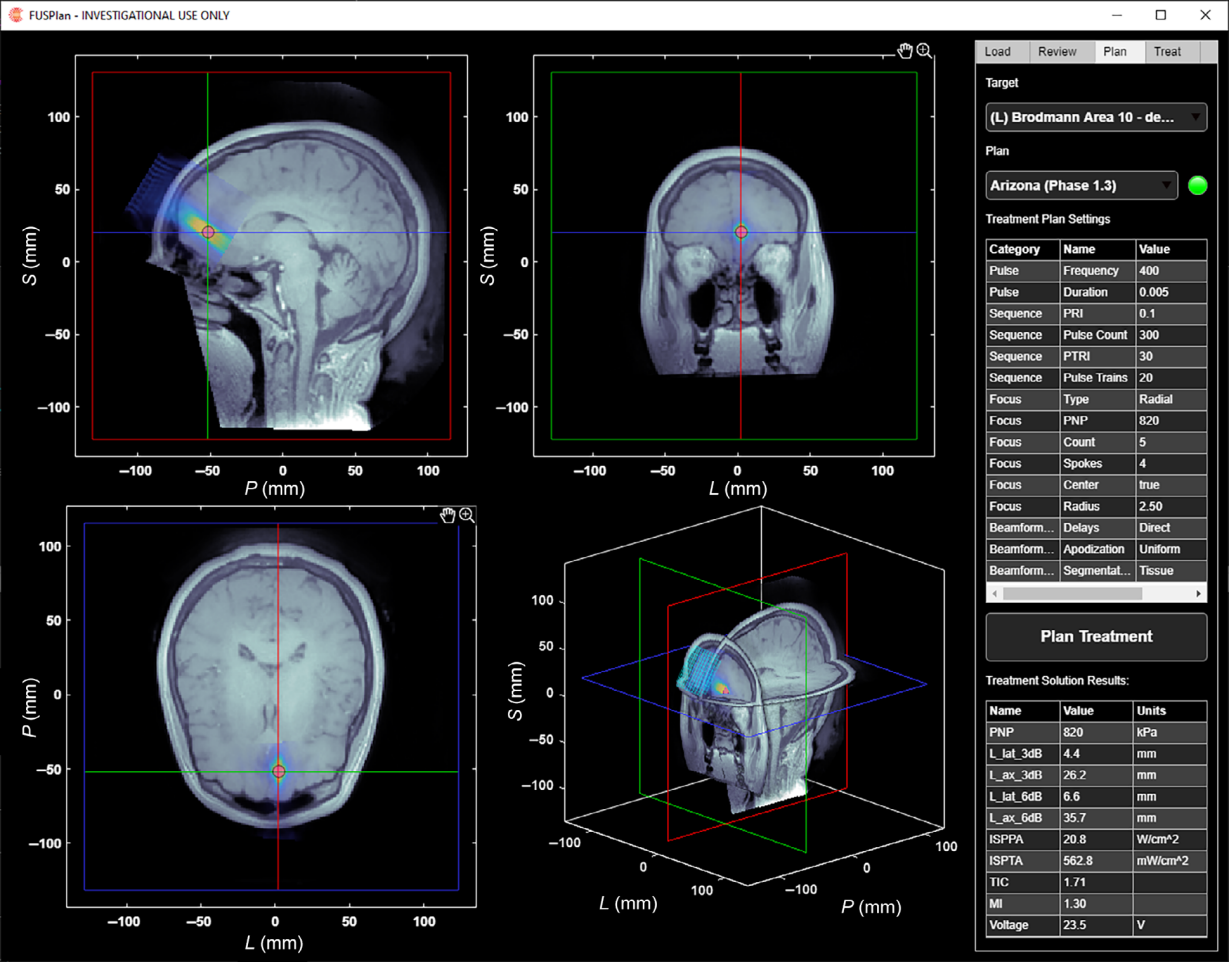
Openwater planning software screenshot. The left side of the image shows the MRI, with the transducer, target, and simulated pressure field overlain. The right side shows the “Plan” panel, containing information about the treatment protocol and analysis of the treatment solution.

To coordinate the planning and delivery of the tFUS beam, a custom MATLAB software program and GUI was developed. The software interfaces with the neuronavigation and driving electronics subsystems to assist with positioning of the headset, compute a beamforming solution for delivering ultrasound from the array to the person‐specific target, simulate the delivered acoustic field, and configure, initiate, and control ultrasound delivery. The ultrasound planning and delivery sequence is outlined in the flow chart of Figure 6 and a screenshot of the software is shown in Figure 7. The details of each step are described below.

##### tFUS Beam Planning

Once the participant is fit with the headset, the software tracks the position of the array via the neuronavigation system and provides near‐real‐time feedback (within 1 second of being updated in the neuronavigation software) on the steering and number of blocked elements, as were computed in the headset placement planning stage. Once the array is secured in place, the software imports the final array position and loads the requested ultrasound parameters. Next, the MRI volume and target are transformed into the transducer array's frame of reference, which will be the simulation space. The imaging volume is then converted to a map of acoustic parameters (via an assumption of uniformity and the use of segmentation). Next, delays are computed to steer the beam to the target(s). Once this initial solution has been generated, it is simulated at a nominal voltage and pressure amplitude using k‐wave to determine the nominal pressure and intensity fields.^24^ The attenuation in soft tissue is assumed to be 0.3 dB/cm/MHz. After simulation, the pressure at the target(s) is compared with the pressure requirements of the plan, and the input voltage and simulated fields are scaled so that the target(s) will achieve the requested acoustic exposure. Finally, characteristic parameters of the acoustic field are determined to ensure that sidelobes and safety parameters are within acceptable limits, and the acoustic fields and computed parameters are displayed interactively on the screen.

For the initial safety and usability study, it is important to note that beamforming was performed neglecting the presence of the skull, assuming that all tissue between the coupling pad and the target was soft tissue. It was decided that this was the most conservative way to ensure that, regardless of the performance of skull‐segmentation methods, no participant could receive more acoustic pressure than the target dose. It is known that the actual dose received at the focus will be lower than the target nominal dose in a subject‐specific way due to the presence of the skull and its variable shape and thickness. The extent of the effects of the skull on the attenuation and aberration of the focus volume were characterized in post‐study activities.

##### tFUS Delivery

Once the user has reviewed the targeting solution, the software will initiate a connection to the driving electronics, configure the hardware to deliver the ultrasound, and launch a control module for the user to initiate ultrasound exposure. During an ultrasound sequence, a progress bar shows how far into the sequence the participant is, and the temperature of the array is monitored and displayed; if for any reason the sequence needs to be halted, the user could abort through the software in less than a second, or by removing the headset.

#### Driving Electronics

The ultrasound driving electronics allow for sending electrical driving signals to each element of the array individually. It is possible to send an identical waveform to each element with a specified time delay (phase difference), and also possible to modify the waveform of each element individually. The waveforms consist of a 3‐level, bipolar square wave signal. The ultrasound driving electronics used in the prototype system is a Verasonics Vantage Ultrasound Acquisition platform (Verasonics Inc, Kirkland, WA). The Verasonics Vantage system is a standard frequency system with a HIFU option that includes an external 1200‐W DC power supply (QPX600DP, Aim‐TTI, Huntingdon, UK) with both DC output channels connected in parallel to allow for greater source current.

### Ultrasound Capabilities

The ultrasound system can deliver a range of ultrasound parameters, and because it uses a matrix array, has additional complexity available associated with steering the beam within its field of view. We define a Plan as a specification for an ultrasound sequence, including dose targets, beamforming methodology configurations, and constraints on the on steering and/or acoustic safety parameters. For an individual participant, the position of the transducer, their prior imaging data, and the location of the target location within their head are provided as inputs to the plan and used to generate a Solution, which contains the delays, voltages, and timings that the hardware needs to deliver the specified ultrasound dose for that particular participant. For the system presented here, the options that define a Plan are shown in Table 1: Available Configuration Parameters. For many parameters, the software is highly modular, meaning that new methods can be programmed and used beyond the system defaults used in the study.

**Table 1:** Available Configuration Parameters

Category	Parameter	Description	Typical	Min	Max	Units
*Pulse*	Frequency	Pulse center frequency	400	N/A	N/A	kHz
*Pulse*	Voltage	Transmit voltage (±)	20	5	35	V
*Pulse*	Duration	Pulse duration	5	0	100	ms
*Sequence*	PRI	Pulse repetition interval	100	10	1000	ms
*Sequence*	Pulse count	Number of pulses per train	300	1	1000	
*Sequence*	PTRI	Pulse train repetition interval	30	0	120	s
*Sequence*	Train count	Number of pulse trains	20	1		
*Beamforming*	PNP	Target pressure	820	100	1000	kPa
*Simulation*	Spacing	Voxel spacing	1			mm

Category	Parameter	Description (Qualitative or Categorical)
*Beamforming*	Shape	Pattern shape and associated parameters
*Beamforming*	Focal spot	@50 mm: 4.5 mm diameter, 25 mm axial extent (−3 dB pressure)
*Beamforming*	Delays	Delay calculation method. Direct or raytraced
*Beamforming*	Apodization	Apodization calculation method (uniform, maximum angle, or piecewise linear)
*Beamforming*	Segmentation	Segmentation Method and associated parameters
*Beamforming*	Materials	Material properties (speed of sound, density, attenuation coefficient, thermal conductivity and specific heat) for bone, tissue, air, water, coupling pad
*Simulation*	Grid extent	Lat‐Ele‐Ax extent (mm). Typical (−30, 30), (−30, 30), (−4, 80)
*Constraints*	Parameter constraints	Configurable constraints to throw warnings or errors that block ultrasound initiation when a computed parameter (such as MI or *I*_SPTA_) exceeds a set value
*Constraints*	Target constraints	Absolute limits on the spatial position of the target relative to the transducer. Target constraints can be used to identify invalid targets before beamforming and simulation.

### System Characterization

#### Acoustic Measurements

##### Acoustic Field Measurements

Characterization of the transducer array's acoustic focusing abilities were performed to determine the size, shape and intensity of the acoustic focuses generated by electronically steering the array. This enabled determination of the beamforming performance of the array and driving electronics to validate electronic steering and simulation accuracy. Measurements were made in a water tank filled with de‐ionized, degassed water at room temperature, using a computer‐controlled 3D positioning system (AIMS, Onda Corp., Sunnyvale, CA). Calibrated hydrophones (HNR‐0500, Onda Corp, Sunnyvale, CA) were used to ensure that measurements were accurate and repeatable.

To determine the voltage to pressure relationship for each of the steering locations, pressure field measurements were performed while the array focus was electronically steered. Steered locations included all of the boundaries of the steered volume with intervals not exceeding 5 mm from location to location within the volume. These measurements allowed for a 3D interpolation of voltage required for a desired pressure at all of locations within the volume. To validate the interpolation, grid spot checking of over 50 locations ensure that the measurements were accurate and repeatable.

##### Acoustic Power Measurements

Acoustic radiation force measurements were performed using a low frequency radiation force balance (Precision Acoustics, Dorchester, UK). These measurements allowed the determining the acoustic power output of the transducer under various driving conditions. The measurements were taken in de‐ionized water at room temperature and the driving conditions were set to match the frequency and apodization of the study parameters but operated in continuous wave mode. Measurements were taken at different driving voltage levels that spanned all of those used during the study. These measurements were then compared with the calculated acoustic power from the simulations.

#### Simulation Validation With Water Tank

To validate the simulation methods used in the software, simulations of the same signals used for the water tank measurements were performed in both k‐wave and FIELD II^25^. The simulated and measured pressure fields were visually compared and the −3, −6, and −12 dB contours were computed for a sample of steering locations within the steering range of the transducer and overlain on one another to determine how well the spatial pressure fields overlap between methods.

#### Establishing Steering Limits

Because the shape and amplitude of the focused beam varies with focal location, the transmit voltage is scaled up to compensate for a loss of focal gain when the target is moved away from the nominal focus. For the presented experiments, a target peak‐negative‐pressure (PNP) at the focus was used to calculate the necessary voltage adjustment. However, using different voltages means generating different surface powers, so thermal heating of the skull (TIC) and other safety parameters become dependent on target location.

A grid of targets, covering −12.5 to 12.5 mm laterally, −20 to 10 mm in elevation, and 40 to 60 mm axially was simulated to cover a reasonably nominal steering range to target the amPFC through the forehead. Within this range, the various beamforming, safety and exposure parameters were calculated for each candidate target location, allowing for the definition of a sub‐region of valid targets for which all parameters met acceptability criteria.

#### Thermal Simulation

Currently, the system does all of the acoustic simulations with the properties of homogeneous soft tissue. Since heat‐related bio‐effects are known not to occur until exposures of 1.5 to 2.5°C above baseline temperature for at least 1 hour, the system limits heating to 2°C and a maximum possible total ultrasound time of 10 minutes.^26^, ^27^ This is also in line with the latest ITRUSST recommendations which recommend no more than 10 minutes of ultrasound if the Thermal Index is 3 or above.^28^ To estimate the level of focal tissue heating associated with ultrasound, k‐wave's thermal simulation toolbox was used, for both nominal and steered (most overlap between multiple foci) conditions. Because the heating was low, it was validated that the results were effectively the same whether they were obtained by simulating the entire ultrasound duration (with all excitations), or simulating each focal location once individually, and superimposing the differential results for each excitation in the sequence. This second method allowed for rapid investigation of different ultrasound sequences. Thermal Index at the Cranium (TIC) was derived directly from the transmitted ultrasound intensity according to TIC = *W*/*D*/*C*_TIC_, where W is the time‐averaged acoustic power in mW, and *D* = 5.31 cm is the equivalent aperture diameter of the array and *C*_TIC_ is 40 mW/cm.^28^

#### Still Air and Simulated Use Thermal Testing

Thermal testing of the array was performed to ensure that it was able to pass the Still Air and Stimulated use tests outlined in the IEC 60601‐2‐37 standard. These tests were performed with the same stimulation parameters as the study (5 ms burst, 10 Hz PRF, same waveform apodization), but at a 20% higher driving voltage than used during the study. There was no off time during the duration of the tests.

The still air test consisted of running the probe at full power while suspended in air with no acoustic coupling of the probe. Measurements were made using thermocouples taped to the face of the probe, thermocouples inside of the probe face, and using a thermal imaging camera (Teledyne FLIR, Wilsonville, OR). The still air test was run for 20 minutes.

For the simulated use test a tissue mimicking material (TMM) phantom was fabricated with a hydrated human skull bone at one side. The TMM was a cube with ~10 cm sides except for the embedded skull bone. A thin layer (1–2 mm) of TMM was poured on the outside of the skull before applying a 1.5 mm silicone sheet placed on top. The ultrasound array was placed in contact with the silicone through the custom polymer coupling pad with ultrasound gel to closely mimic the use case. Thermocouples were placed in the center of the array between the outside of the skull bone and the TMM to simulate heat of the skull, and between the silicone sheet and the coupling pad to simulate heating of the skin. The thermal study was run for 30 minutes (triple the maximum ultrasound time).

### Human Participant Protocol to Assess Safety and Useability

#### Study Overview

The neuromodulation system safety and useability study used the system to deliver tFUS to human participants and assess system usability, monitor adverse events (AEs), and to provide a neuroradiological read of post‐delivery susceptibility‐weighted images that can probe for vascular micro‐hemorrhages. The study was broken up into three sub‐“phases” which gradually increased the intensity and complexity of the LIFU protocol across each phase until the final phase, which would represent the paradigm used in subsequent research. The Institutional Review Board of the University of Arizona approved the experimental protocol. All participants signed an IRB‐approved informed consent document before participation.

#### Participants

Twenty individuals (16 F, 4 M) participated in this study. Participants recruited for this study were selected to have some degree of repetitive negative thinking, having a score on the Perseverative Thinking Questionnaire (PTQ)^29^ above the 25th percentile. Inclusion criteria also included age 18–35, English‐speaking, and without any neurological symptoms or symptoms of mania/psychosis. Exclusion criteria included: smoker or use of tobacco products or any form of nicotine daily or nearly daily, history of head injury with loss of consciousness for more than 5 minutes, uncorrected vision and/or hearing impairment, current or history of brain or mental illness judged likely to interfere with testing, including drug and/or alcohol dependence, a diagnosed sleep disorder (eg, Insomnia), current drug, alcohol, or prescription drug intoxication, history of epilepsy, history of migraines, metal implants in head or face (including permanent dental retainers), history of cardiac problems, current major depressive disorder, a score higher than a 24 on the Beck Depression Inventory II (BDI‐II),^30^ and acute suicidal ideation.

Additionally, participants were excluded from the experiment if they were pregnant or unsure if they may be pregnant or have any contraindications for MRI, including severe claustrophobia, non‐MRI compatible cardiac pacemakers; implantable defibrillators; aneurysm clips; neural stimulators; artificial heart valves; ear implants; insulin pumps; drug infusion devices; IUDs; magnetic dental appliances; metal fragments or foreign objects in the eyes, skin or body; metal plates, screws, and prosthetics; non‐removable metal piercings; tattoos on the head and neck, other certain older tattoos or permanent makeup (eyeliner) using metal‐containing inks, some medicated patches, metal implant or other injury or device that is contraindicated for MRI.

Participants were recruited through two methods, the Psychology department introductory psychology participant pool and via flyers in the community. Following consent, participants completed screening questions and the PTQ and BDI‐II, followed by MRI scanning, tFUS delivery, and more MRI scanning. The entire study took about 2 hours to complete.

##### Participant Demographics

Participants' mean age was 23.6 (SD = 6.21) and 80% were women (16/20, four identified as male), with 80% identifying as white (16 White, 2 Asian, and 2 Undeclared) and 70% identifying as non‐Hispanic (14 Non‐Hispanic, 4 Hispanic, 2 Undeclared). The mean PTQ score was 33.1 (SD = 6.72) and the mean BDI‐II score was 11 (SD = 5.58).

#### Study Protocol

Before ultrasound delivery, participants completed an MRI session including a structural T1 scan and a 3‐minute PETRA short TE scan to assess skull thickness and density. The ultrasound was administered immediately after completion of the baseline MRI. Focused ultrasound targeted the left anterior medial Prefrontal Cortex (amPFC; Talairach coordinates in Table 2). The amPFC is a core hub of the default mode network (DMN) that subserves self‐relevant thought,^31^ and was selected for a future study examining tFUS in major depression, a condition characterized by hyperconnectivity within the DMN.^32^ Given the focus on linguistic repetitive negative thought for that future study, the left hemisphere amPFC was favored over the right as the target.

**Table 2:** Ultrasound Target and Focusing Properties Used in Each “Phase” of the Study.

Phase	N	Target Description	MNI Target Location	PNP (kPa)	MI	*I*_SPTA_ (mW/cm^2^)
1.1a	3	(L) Brodmann Area 10	−6, 52, −2	510	0.8	435
1.1b	6	(L) Brodmann Area 10	−5, 45, −3	510	0.8	435
1.2	6	(L) Brodmann Area 10 (multi‐focus)	−5, 45, −3	650	1.03	<450
1.3	5	(L) Brodmann Area 10 (multi‐focus)	−5, 45, −3	820	1.3	<675

*Note*: The MNI target location is specified using the RAS (right‐anterior‐superior) coordinates in cm within the Montreal Neurological Institute (MNI) brain atlas, before registration to each individual's brain geometry.

The ultrasound delivery parameters (ie, frequency, pulse length, and pulse repetition frequency) remained the same throughout the study, but the pressure amplitude was systematically varied in increments to optimize the probability of an effect and ensure safety. In three discrete phases, the maximum pressure and spatial‐peak, temporal‐average intensity (*I*_SPTA_) did not exceed diagnostic levels. Additionally, the target location and the dosing scheme were adjusted to target a larger area and increase the likelihood of an effect while maintaining safety and useability, as described in Section 1.4.5.

After the ultrasound device was placed on each participant's forehead with the custom headset and all parameters were established by operators trained in the use of the neuronavigation system, participants were instructed to sit quietly and keep their eyes open for the duration of the ultrasound delivery, which was 10 minutes. It should be noted that the “virtual fitting” step described in Section 1.5.2 was developed during the study (in response to the discovery that some subjects had pronounced sinuses), so was not used during headset fitting, but instead used retrospectively to compare the unguided transducer placement to the “optimized” positions.

After ultrasound delivery, participants completed another MRI scanning session which included a 5‐minute Susceptibility‐weighted image scan to check for any signs of possible injury to the brain.

#### Magnetic Resonance Imaging Parameters

Two imaging sequences were used to acquire structural MRIs (T1 and PETRA)^33^ that allowed for the identification of the prefrontal cortex target and estimate skull thickness and sinus location for acoustic simulations, and for registration with the neuronavigation system for targeting. All images were acquired on 3 Tesla Siemens Skyra scanner (https://www.siemens-healthineers.com/en-us/magnetic-resonance-imaging/3t-mri-scanner/magnetom-skyra), which is a standard research‐grade MRI system.

T1‐weighted anatomical images were acquired for registration of the functional scans and to assist in the delivery of the ultrasound. (MP‐RAGE; TR = 2100 ms; TE = 2.33 ms; TI = 1100 ms; flip angle = 12; FOV = 256 mm; acquisition voxel size 1 mm × 1 mm × 1 mm).

PETRA anatomical images were acquired for registration of the functional scans and to assist in the delivery of the ultrasound. (TR 1 = 5 ms; TE = 0.07 ms; flip angle = 6; FOV = 240 mm; acquisition voxel size 0.9 mm × 0.9 mm × 0.9 mm).

Susceptibility‐weighted images (SWI) were acquired to assess safety of ultrasound delivery. (TR = 28 ms; TE = 20 ms; flip angle = 15; FOV = 220 mm; acquisition voxel size 0.6 mm × 0.6 mm × 1.5 mm).

#### Ultrasound Application and Parameters

While the simulation results indicated the ultrasound beams were below the safety thresholds for diagnostic ultrasound, a conservative stepped approach was taken with volunteers. In Phase 1.1 of the study, consisting of the first nine participants, a single low peak negative pressure (PNP) amplitude (510 kPa) focus was used. Following no AEs, the PNP for each focus was increased to 650 kPa for the six participants in Phase 1.2. In addition to changing the focal pressure a new multi‐focus approach was implemented that increased the effective area where the ultrasound energy was delivered as detailed below. Following no AEs in Phase 1.2, Phase 1.3 ultrasound parameters were changed again by increasing the PNP of the pulses to 820 kPa, while still keeping the new multi‐focus approach of interleaved pulses. This was done to both maximize the ultrasound area and pulse pressure.

**Figure 8:**
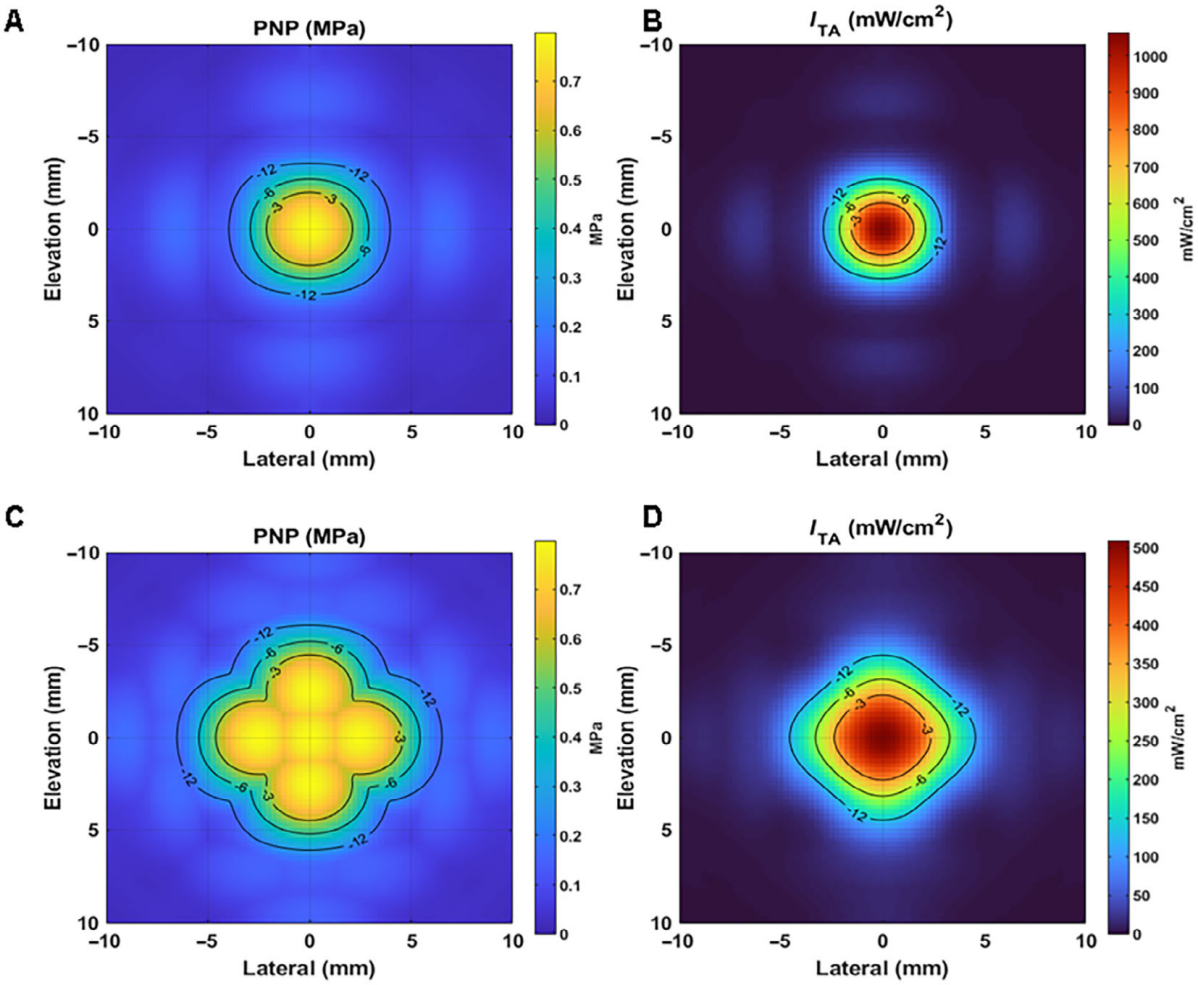
**A** and **B**, Lateral‐elevation profile of single nominal focus. **C** and **D**, Lateral‐elevation profile of focal pattern used during last Phases 1.2 and 1.3 of the safety study. **A** and **C**, Pressure field with −12, −6, and −3 dB contour lines. **B** and **D**, Temporal average intensity −12, −6, and −3 dB contour lines.

**Figure 9:**
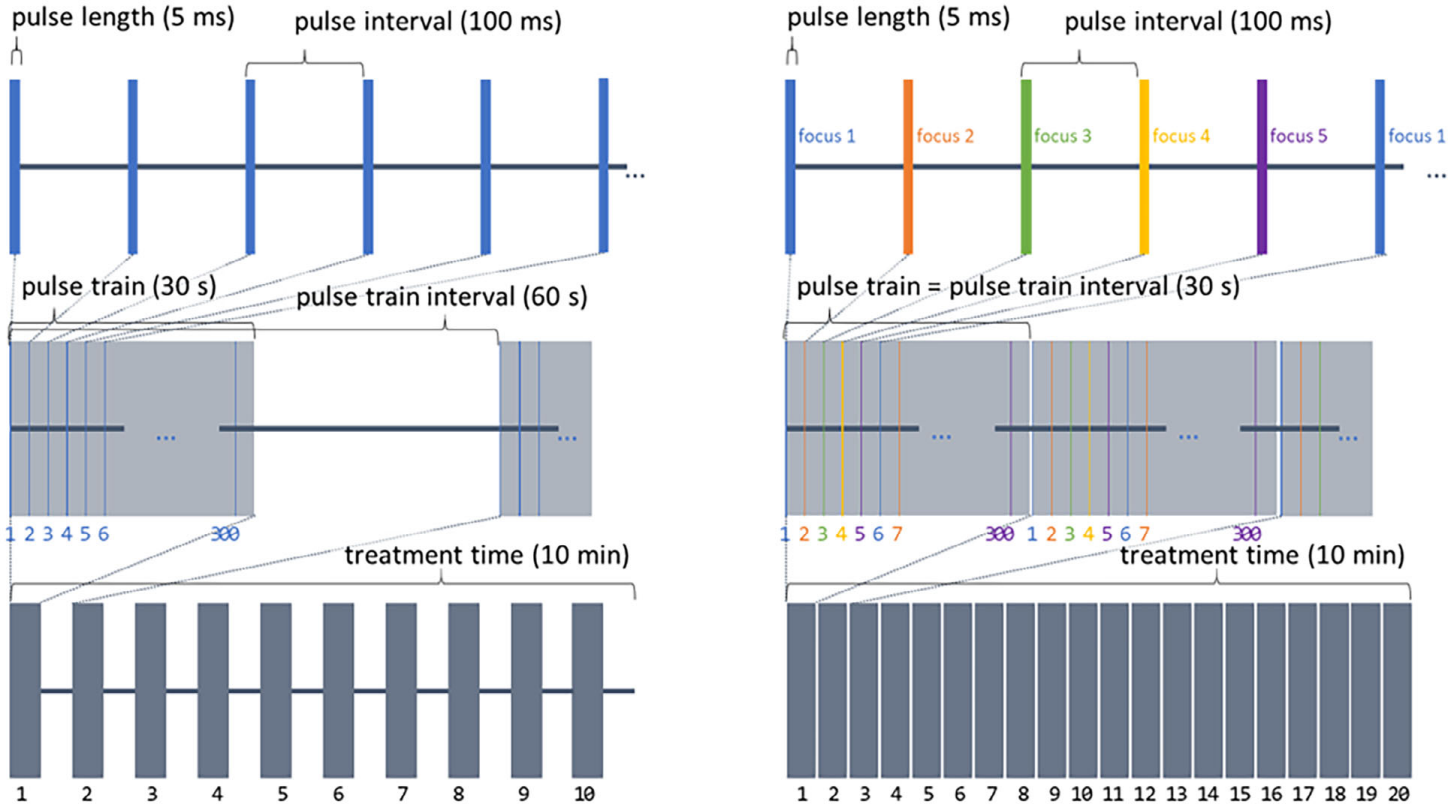
Pulse sequences. Left: Phase 1.1; Right: Phase 1.2 and Phase 1.3. In Phase 1.1, a single focus is used, with a 30 seconds on–off pattern of pulse trains. In the later phases, the pulses are rastered across five partially offset foci, resulting in a longer per‐focus PRI but allowing greater spatial coverage and requiring no on‐off cycling.

The new multi‐focus approach (termed interleaved pulses) consisted of targeting multiple foci, one after another (repeating). A radial focus was used (as shown in Figure 8) was used, which positioned five foci in a cross‐like radial pattern. The distance of the four foci position around the central focus was 5 mm, which corresponded to the width of the focus (−6 dB pressure) when steered to the nominal location 50 mm from the center of the face of the transducer array. Each pulse was applied individually but in sequence. This method of pulse interleaving is visually represented in Figure 9. It allowed for spatially distributing the energy delivery in the target region providing a means of increasing the PNP without increasing the *I*_SPTA_ experienced at any given focus.

The pulse sequences for each phase are visualized in Figure 9.

### Post‐Study Development

Upon the completion and review of the safety study results, it was noted that in some subjects, the path of the ultrasound beam passed through the sinus cavity. To explore this more deeply, additional post hoc analysis was performed.

#### Sinus Analysis

**Figure 10:**
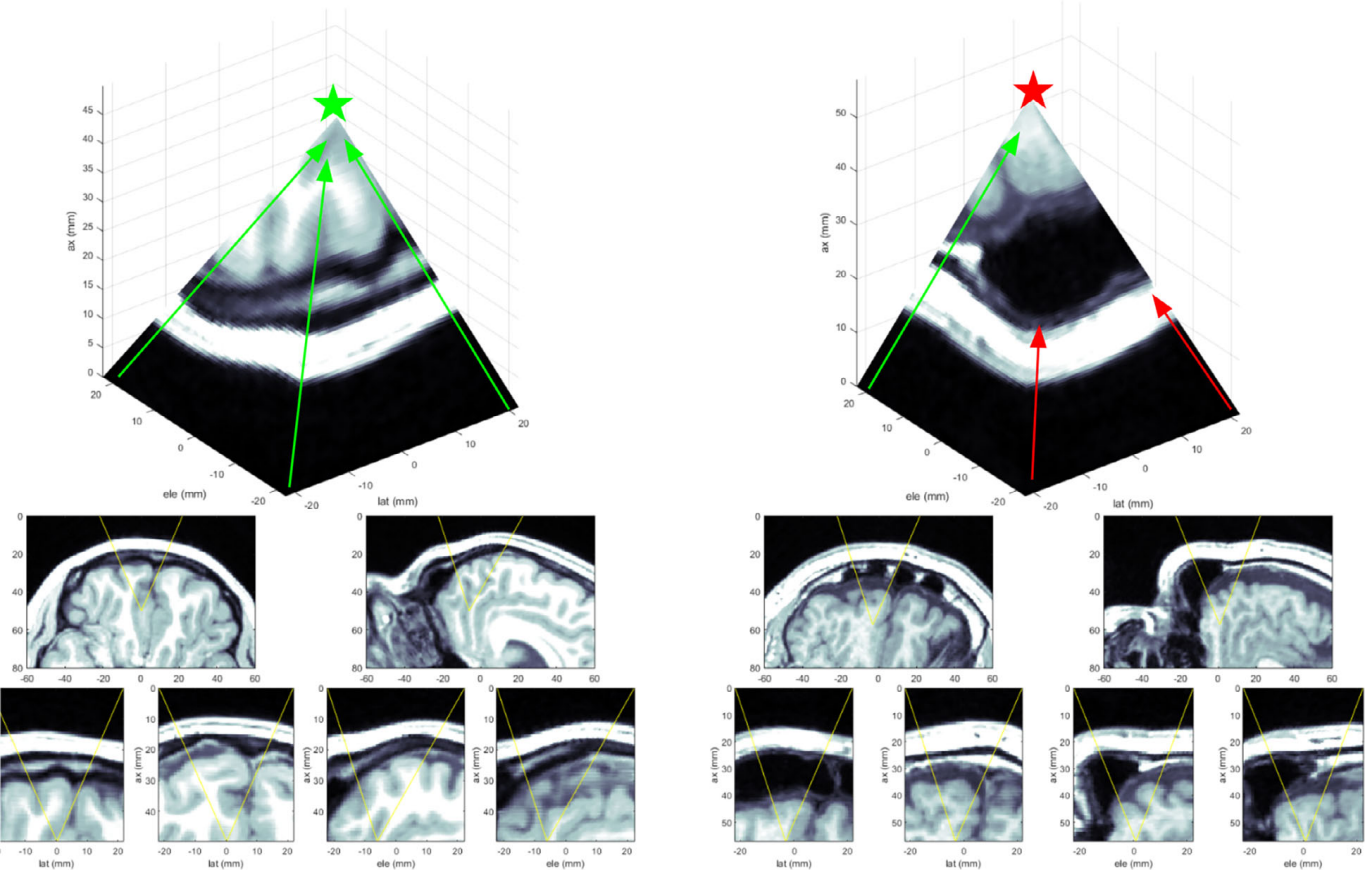
Blocked sinus comparison. Left: An unobstructed path to the target. Right: a path obstructed by the sinuses.

The first post hoc analysis was to evaluate which of array elements passed through the sinus cavity. To do this, a simple thresholding scheme was implemented. The MRI data between each element of the ultrasound array and the patient specific target are analyzed as a “pyramid” of rays (Figure 10). Detection of the sinuses is performed as follows:

1. Trace rays with equal step sizes from each element to the target.
2. Normalize the brightness of the ray so that the average of the first 1 mm of all rays is set to 0 (air the head), and the 90th percentile of the last 2 mm of all rays is set to 1 (white matter).
3. For each normalized ray, determine if any voxels fall below 10% of the white matter brightness.

For each subject and transducer position, the number of blocked rays was calculated in this way.

#### Development of Virtual Fitting

**Figure 11:**
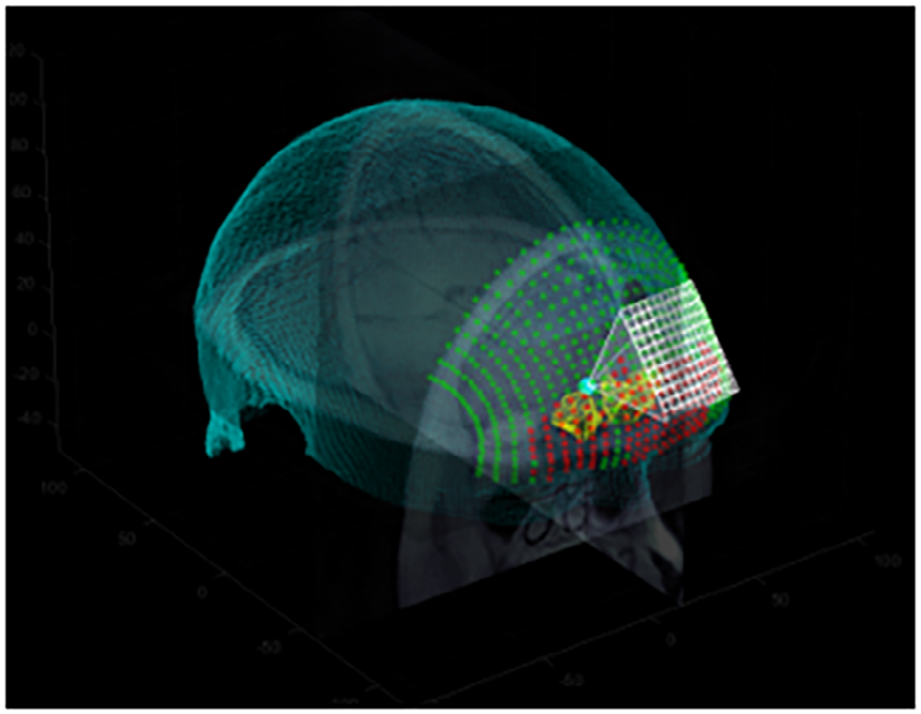
Visualization of sinuses and probe placement. The green dots represent entry points along which sinus obstruction was evaluated, the yellow volumes are the segmented sinuses, the cyan dot is the target, and red dots represent entry points for which the path was determined to be obstructed.

To aid the positioning of the array, a method was developed to recommend an optimal location. The algorithm optimizes the placement by minimizing the distance between the array and the target while ensuring that the target is within the acceptable steering range of the array (defined in Section 1.3.3), and by minimizing the presence of air‐filled sinuses located between the array and the target that will block the ultrasound delivery. The optimization is performed as follows:

Once the person‐specific target location has been defined in the person's anatomical MR scan (as described in the prior section), the MR volume and target location are imported into the software. The outer surface of the skin is automatically segmented via simple thresholding to provide a surface map in polar coordinates. Next, a series of candidate positions (between 10 and 40 degrees above the eye‐line in three degree increments, and between 5 degrees to the subject's right eye and 25 degrees toward their left ear from the center‐line = 121 positions) for the ultrasound array are defined. For each position, the corresponding subsection of the skin surface is fit to a plane to determine the approximate normal vector to the skin surface. The known transducer geometry is then virtually placed relative to the skin surface, offset to account for the wedged shape of the coupling pad. In each such candidate position, the rays between each element and the target are analyzed as described in Section 1.5.1, as well as the nominal steering required.
Once the steering and number of blocked elements has been determined for each position, candidate positions whose steering is outside the valid steering range for the probe are excluded, and then the remaining points are filtered to those with the lowest decile of blocked elements. From those final candidates, the “optimized” position is then chosen as the one closest to the target. This position, along with maps of blocked elements and steering validity, are presented to the user, overlaid on the detected skin surface, to provide a visual guide for where to place the probe, as shown in Figure 11.

#### Segmentation and Re‐Simulation

We evaluated the extent to which the focal spots were distorted within participants, both in terms of amplitude, as well as steering. The defaced T1 and Petra scans were manually segmented using a segmentation service, the results were reviewed and approved by the team, and used to run a second set of simulations for each subject. These simulations used the same amplitude and delays as were run in the actual study but included heterogeneous maps of acoustic parameters per the simulation result. The materials and acoustic properties used in the simulations are listed in Table 3.

**Table 3:** Material and Acoustic Properties Used in the Segmented Simulations

Material	Speed of Sound (m/s)	Density (kg/m^3^)	Attenuation (dB/cm/MHz)
Water	1500	1000	0.0022
Soft tissue	1540	1050	0.3
Skull	2800	1900	6
Coupling pad	1420	1000	1
Air	344	1.25	7.5

## Results

### System Characterization and Validation

#### Simulation Validation

**Figure 12:**
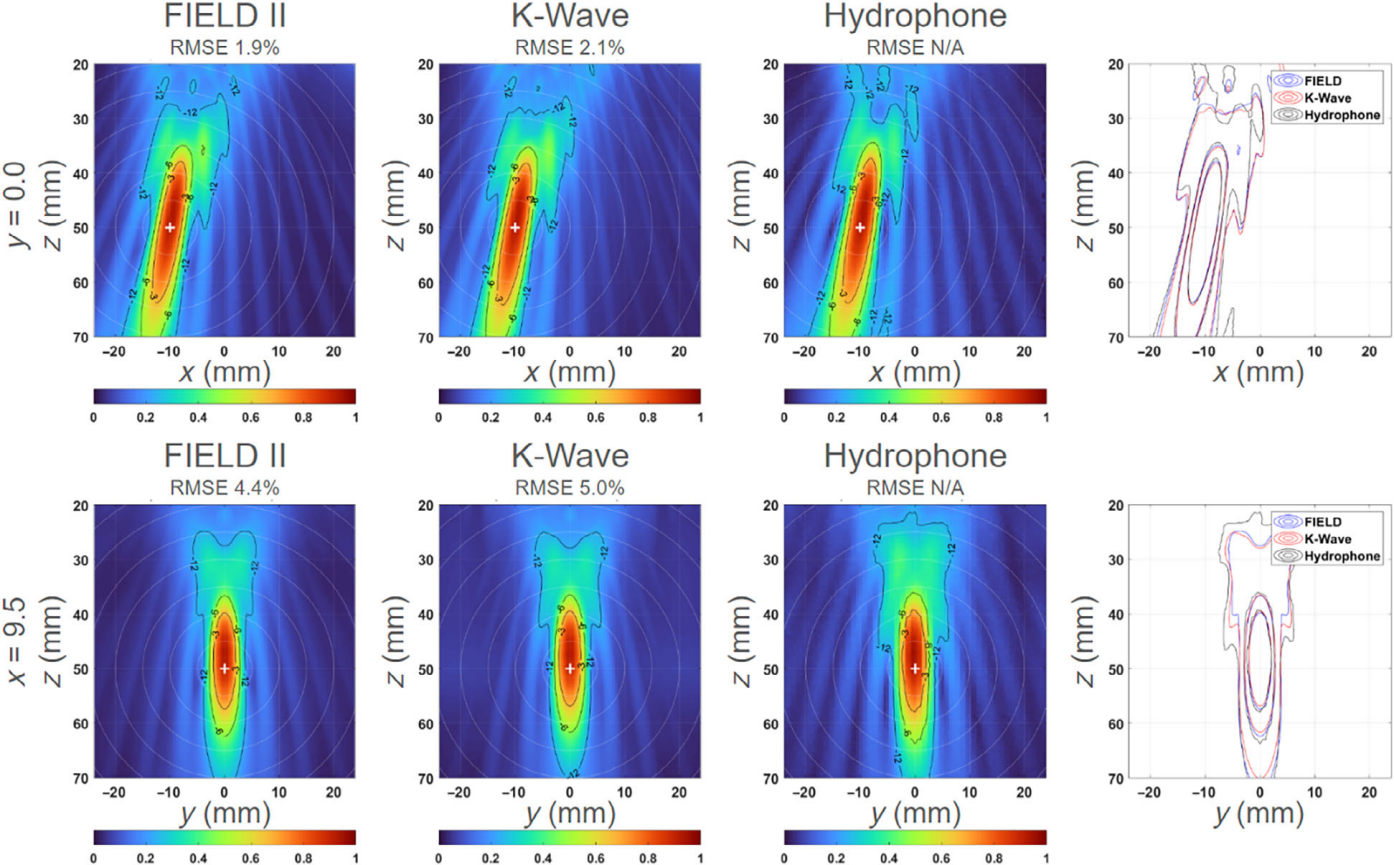
Comparison of simulated and measured beamplots. The top row shows *x*‐*z* cross‐sections, and the bottom row shows *y*‐*z* cross sections.

Cross sections of one of the matched hydrophone scan volumes are shown in Figure 12. The leftmost column shows FIELD II results in the *xz* and *yz* planes for a purely water medium, normalized to their global maxima. The second column shows k‐wave results, the third column shows Hydrophone results, and the final column shows the −3, −6, and −12 dB contours for each of the three methods. Each slice is also labeled with the root‐mean‐squared‐error (RMSE) of the pressures between the slice and the hydrophone data as a percentage of the peak. Good qualitative agreement between the contours is seen, and the distribution of pixel values shows little difference in the shape of the beams. Most visual differences are well below the −12 dB value, and the RMSE values indicate agreement within 5% between the two simulation methods and the hydrophone measurements.

#### Acoustic Output Results

The results of simulating each focal target in the 20 volunteers are shown in Table 4, grouped by the three different ultrasound sequences used in each phase. The simulations were performed assuming homogeneous soft tissue. The target focal positions describe where, within the transducer's field of view, each target lay. While operators aimed to get the target as close to 0, 0, 50—the nominal focus of the transducer, as possible, all targets fell well within the matrix array's steerable field. For some parameters (PNP, ISPPA, ISPTA, and MI), the values are directly determined by the sequence and pulse parameters and independent of the focal position. One notable exception is ISPTA for Phase 1.3, because the shapes of the different foci vary slightly with focal position, resulting in different contributions to the time‐average of the spatial‐peak voxel. The −3 and −6 dB beamwidth parameters describe the shape of the focal region, which has a characteristic “cigar” shape with a longer axial depth of field than lateral or elevation.

**Table 4:** Beamforming and Safety Metrics From K‐Wave Simulation, Covering the Distribution of the Location of the Focal Targets, the Nominal Focal Pressure and Intensity, the Simulated Focal Pattern Shapes, and the Thermal Index (Cranium) and Mechanical Indices

Variable	Phase 1.1	Phase 1.2	Phase 1.3	Units
Number of volunteers	9	6	5	
Target focal position (Lateral)	−0.599 ± 2.3	−2.31 ± 3.2	0.119 ± 2	mm
Target focal position (Elevation)	−4.11 ± 7.1	−4.47 ± 4.1	−1.14 ± 5.4	mm
Target focal position (Axial)	50.7 ± 3.6	52.3 ± 2.6	48 ± 4.2	mm
Peak negative pressure (PNP)	510	650	820	kPa
Pulse‐average intensity (*I*_SPPA_)	8.04	13.1	20.8	W/cm^2^
Time‐average intensity (*I*_SPTA_)	201	327	508 ± 46	mW/cm^2^
Lateral‐elevation (−3 dB) beamwidth	4.32 ± 0.26	4.29 ± 0.19	8.64 ± 0.54	mm
Axial (−3 dB) beamwidth	24.5 ± 2.6	25.3 ± 2	23 ± 2.9	mm
Lateral‐elevation (−6 dB) beamwidth	6.43 ± 0.42	6.28 ± 0.49	10.7 ± 0.66	mm
Axial (−6 dB) beamwidth	34.3 ± 1.8	35.3 ± 1.2	33.1 ± 3.3	mm
Thermal index (TIC)	0.325 ± 0.02	0.536 ± 0.036	1.48 ± 0.22	
Mechanical index (MI)	0.806	1.03	1.3	

#### Steering Results

**Figure 13:**
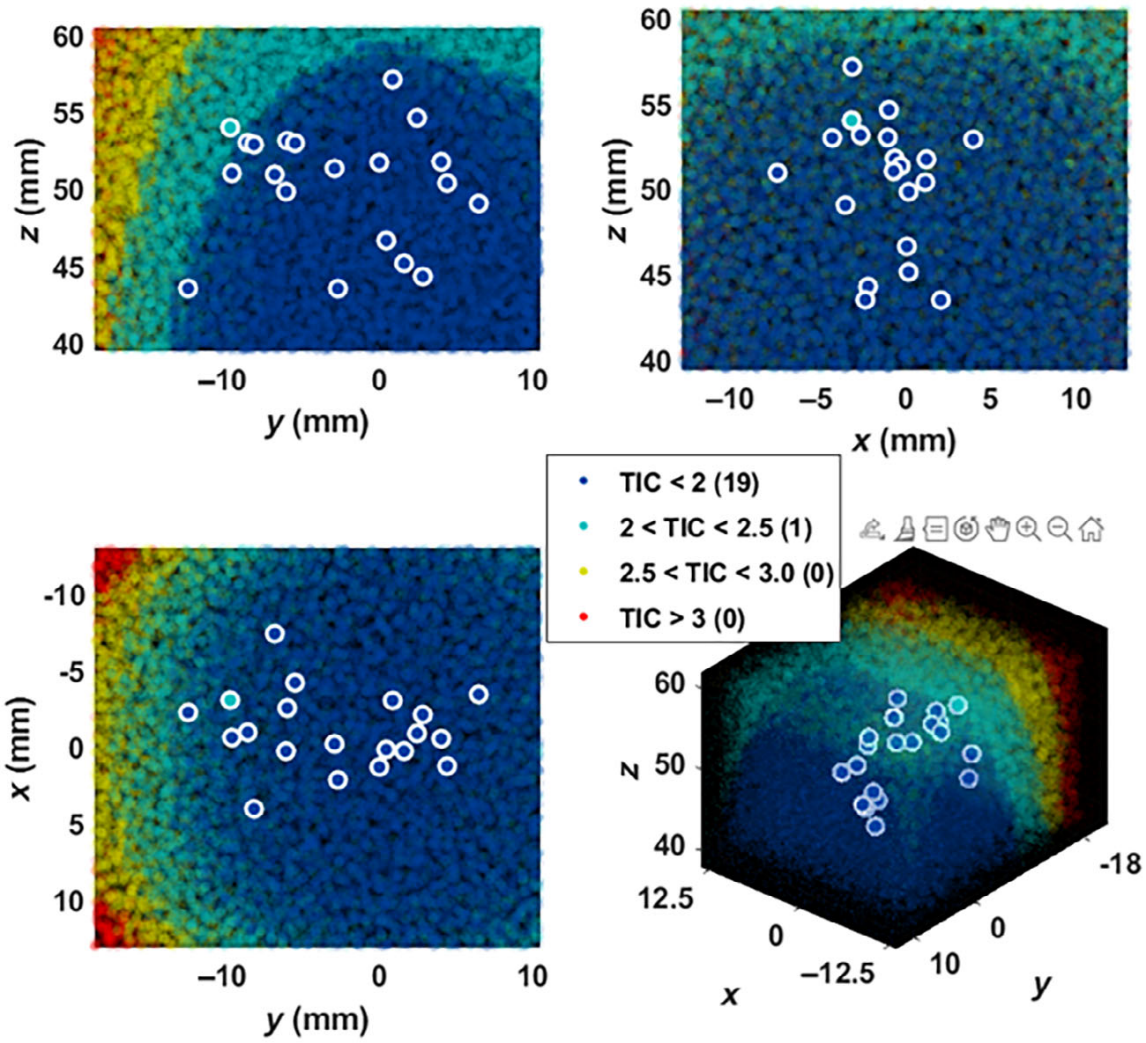
Steering volume represented by TIC value. The positions of the subject‐specific foci are shown circled in white. In general, foci was clustered near the nominal focus (0, 0, 50), meaning that they could be reliably targeted without using gain‐compensatory intensities that would create unacceptable heating in the skull.

TIC was the limiting factor on the ability of the system to steer to targets with higher pressure, because the limited steerability of the array required scaling up the transmit voltage (and thus emitted power) to compensate for decreased sensitivity. The results of the volunteer targets are shown in Figure 13, overlain onto the background TIC volume for the most aggressive ultrasound plan (Phase 1.3). The results indicated that no targets had exceeded a TIC of 2.5 (no points are located in the yellow regions), which means all participants were well within the TIC <3.0 safety limit for a 10‐minute ultrasound. Across all 20 participants, target locations were distributed as follows: lateral (*x*): −0.9 ± 2.6 mm, elevation (*y*): −3.5 ± 5.8 mm, axial (*z*): 50.5 ± 3.7 mm.

#### Still Air and Simulated Use Thermal Results

**Figure 14:**
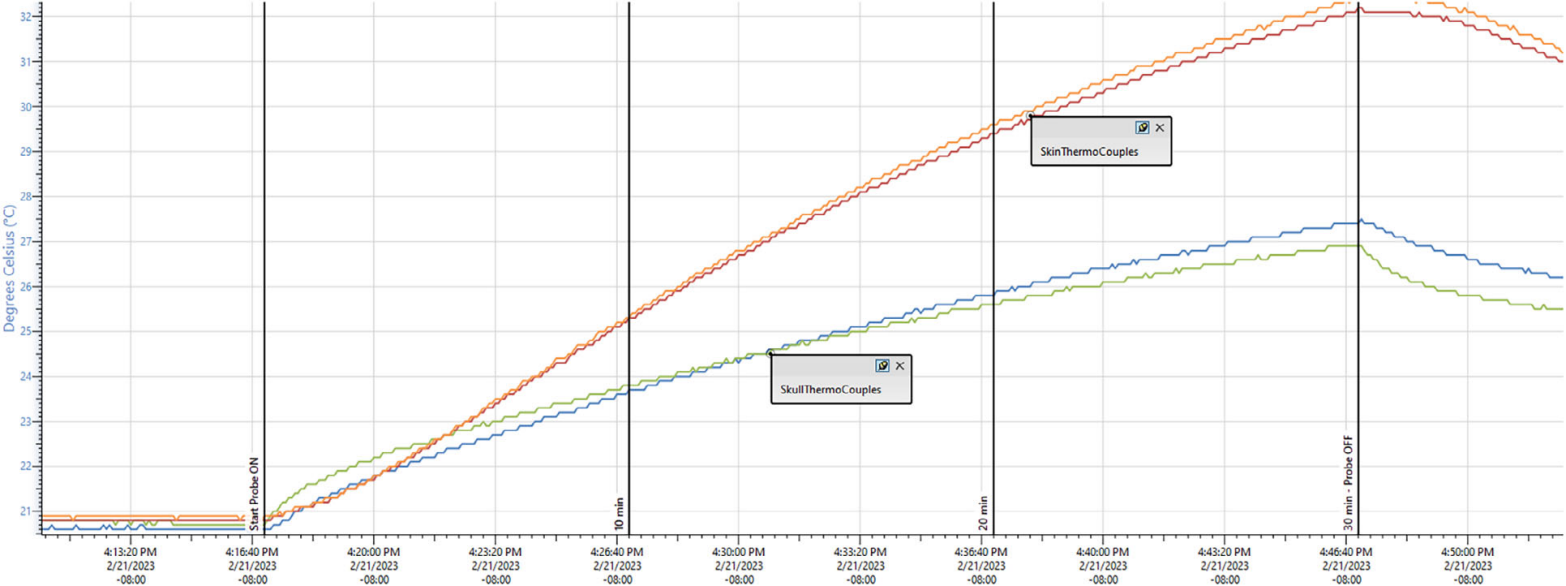
Simulated use thermocouple results. Blue and green are the temperature at the bone surface. Orange and red are the temperature at the skin surface. At 10 minutes (the LIFU duration in the study), the skin surface in the phantom has heated to 25.2°C, and the bone surface to under 24°C.

**Figure 15:**
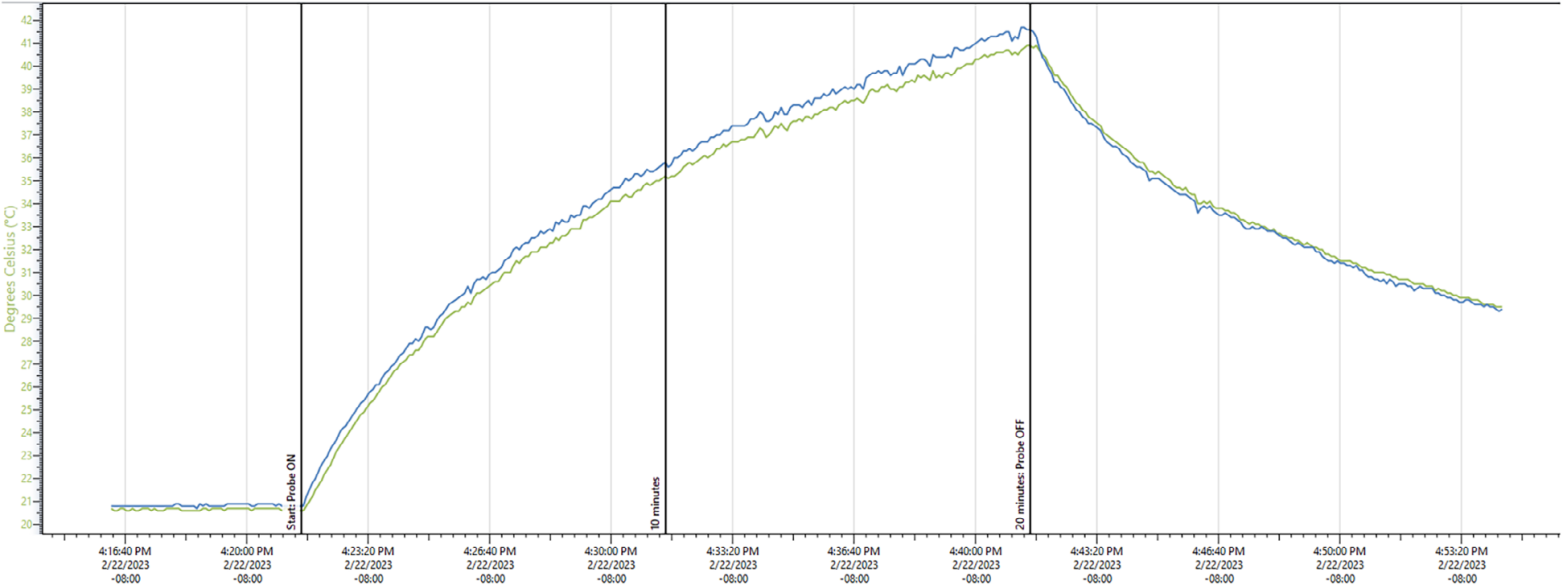
Still air thermocouple results. Blue and green are on the face of the transducer. The temperature reaches 42° at 20 minutes of run time into still air (no coupling material). At 10 minutes (the LIFU duration for the study), face temperature is between 35° and 36°C.

The results of the thermocouple tests are shown in Figures 14 and 15, showing that at no time did the surface exceed 43°C. Into still air, the face of the transducer would exceed 40°C around 20 minutes, which was twice as long as a treatment session, so even an entire session run erroneously into air would not make the transducer face dangerously hot. Heating of the skull surface was estimated from these measurements to be under 3°C from baseline after 10 minutes. Heating of the skin surface was estimated to be 4.2°C. These tests both passed indicating that in the worst case (highest driving voltage) scenario, there was no risk of thermal damage to any participant with the probe in contact with their skin.

### Preliminary Safety/Feasibility Study

#### Usability and Tolerability

Operators of the device reported that the system was easy to use and that the interface was effective at guiding the operation of the device. It was also reported anecdotally that all the participants found the headset tolerable and some of the participants even found the sessions themselves relaxing.

#### Absence of Adverse Events

No serious or subjective AEs were reported from subjects. In addition to monitoring AEs by verbally asking whether subjects experienced any side effects since the last session, a “sensation” questionnaire was administered after each session to assess potential sensations subjects may experience (on a scale from 0 to 10, with 0 being no sensation to 10 being a high amount of sensation) from the ultrasound, including: itching, heat/burning, tingling, vibrating/pulsing, sound, tension, and pain.

For all sensations, the modal response was 0 (no sensation), as shown in Table 5. Importantly, for aversive sensations very high rankings were absent: Burning/Heat max = 5, Pain max = 2. For pain specifically, the three individual reports of pain (ratings of 1 and 2) were reported to be related to the comfort of the headset. Overall, both subjective and objective safety measures indicate that ultrasound was safely delivered and well‐tolerated by participants.

**Table 5:** Sensations During Ultrasound Session

Sensation	Max	Mode	Median	Mean
Itching	4	0	0	0.40
Heat/burning	5	0	0	0.60
Tingling	8	0	1	2.60
Vibrating/pulsing	8	0	1	2.10
Sound	9	0	0	2.15
Tension	8	0	0	1.60
Pain	2	0	0	0.25

#### SWI Imaging

SWI images that were acquired ~30 minutes following the completion of the delivery of tFUS were read by two board‐certified neuroradiologists. SWI images are sensitive to vascular micro‐hemorrhages. All 20 scans were determined to be normal with no findings on SWI, indicating that there were no microhemorrhages.

#### Post‐Study Sinus Analysis

Using the virtual fitting method developed after the study, the acoustic paths for the “optimized” transducer position and the “placed” transducer position were analyzed, and are compared in Table 6. One participant did not have a PETRA scan; thus, the table presents data for 19 of the 20 participants. The position optimization indicated that eight participants had no sinus blockage of the ultrasound beam. Another eight subject had some blockage, but the probe could have been moved to locations with no sinus blockage. Out of the three participants where the sinuses could not be completely avoided, two could have had <20% blockage of the beam path, and only one had sinuses so large that >30% of the beam would be blocked for any placement of the probe.

**Table 6:** Number of Participants With Different % of Blocked Elements for the Used and “Optimized” Transducer Placements

Blocked Elements	None	<10%	10–20%	20–30%	>30%
Actual placement	8	4	3	0	4
Optimal placement	16	1	1	0	1

#### Segmentation and Re‐Simulation

**Figure 16:**
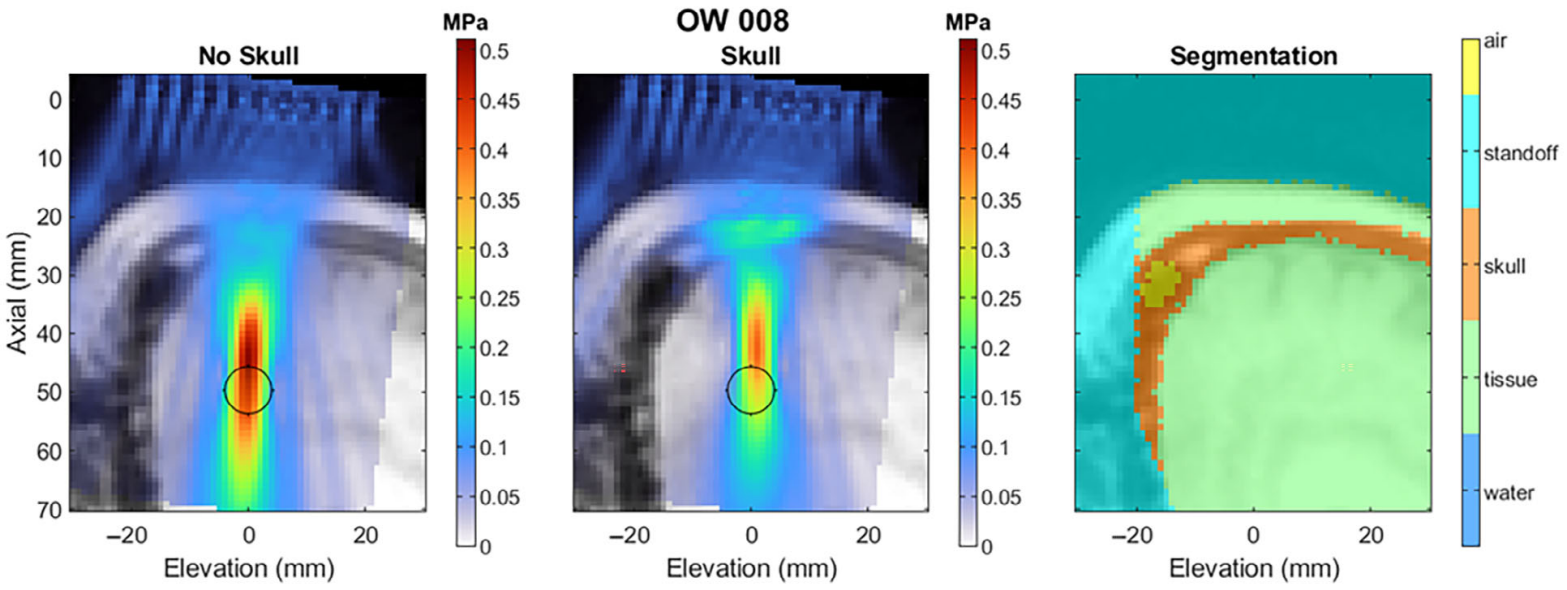
Example segmentation and re‐simulation of a subject with a favorable skull geometry.

**Figure 17:**
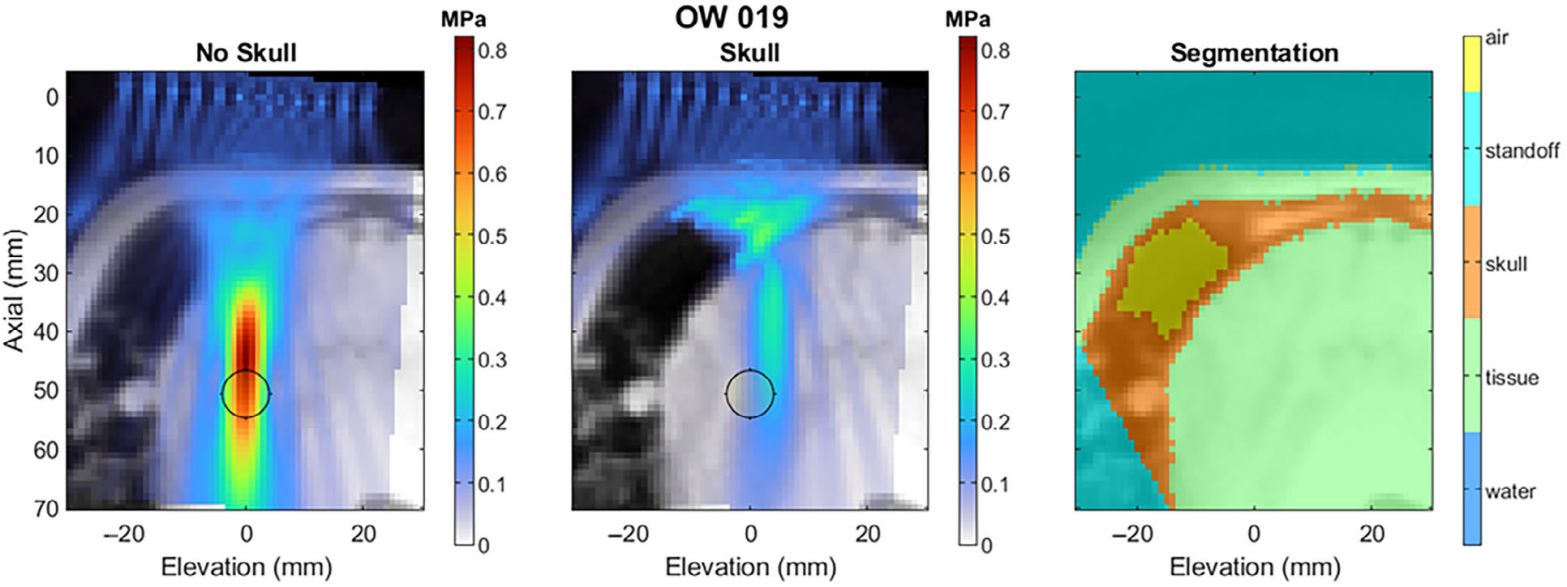
Example segmentation and re‐simulation of a subject with an unfavorable skull geometry.

Figures 16 and 17 show examples of the manually segmented tissue layers and the impact of using the segmentation in the simulation, keeping the delays and amplitudes constant from the “No Skull” case, which matches what was used in the study. Figure 16 shows one of the more favorable skulls, while Figure 17 shows one of the least‐favorable skulls in terms of the level of distortion on the beam. In both cases, the focus is shifted close the transducer, and in the less favorable case, the pronounced nasal cavity obscures a substantial portion of the beam path. In both examples, the segmentation data are cropped due to the defacing method used before sending the data to the segmentation vendor, but the cropped voxels do not appear to be within the beam path.

**Figure 18:**
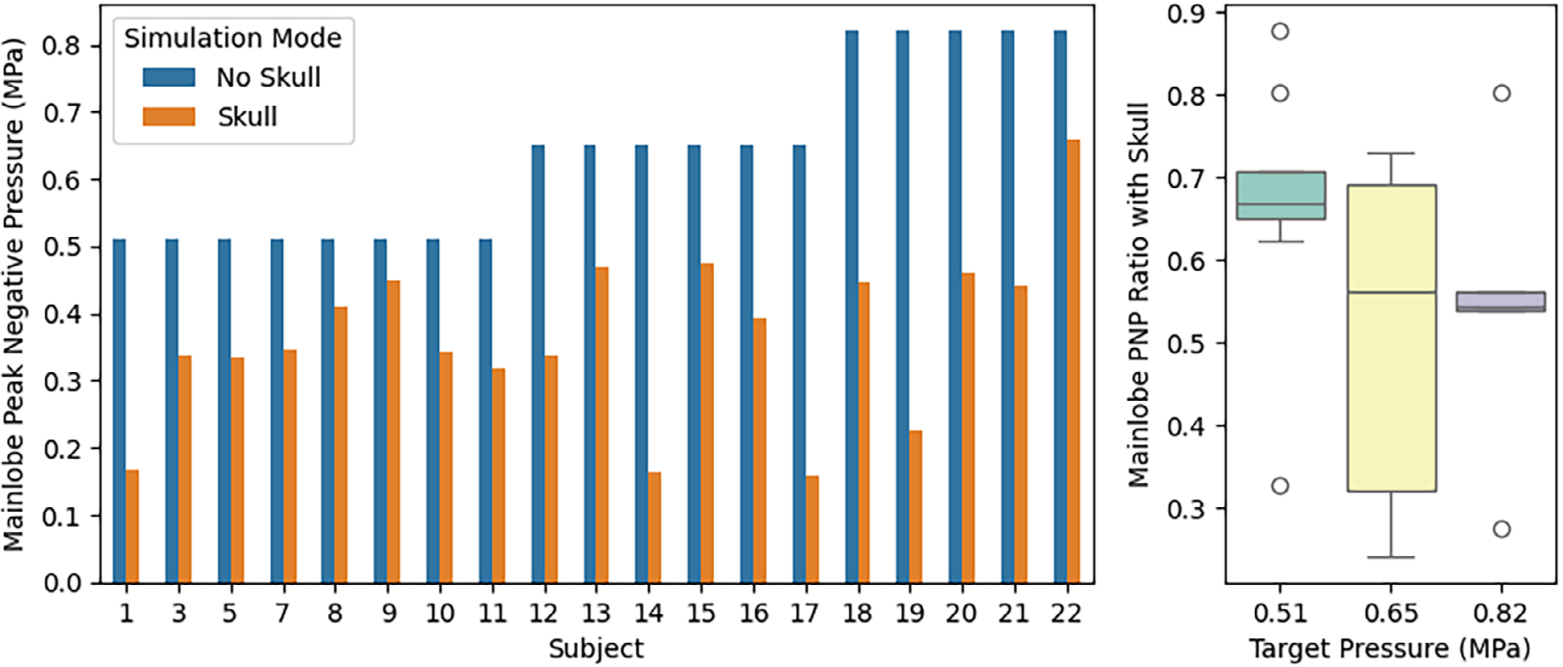
Simulated peak negative pressure with and without the skull. In the “No Skull” cases, the pressure is inherently identical to the nominal target pressure.

Figure 18 shows the drop in peak negative pressure for all subjects. For subjects with multi‐focal beamforming patterns, only the center focus is shown for simplicity. The peak pressure delivered to the target through the skull was 58 ± 19% of the intended target across all three target pressures.

**Figure 19:**
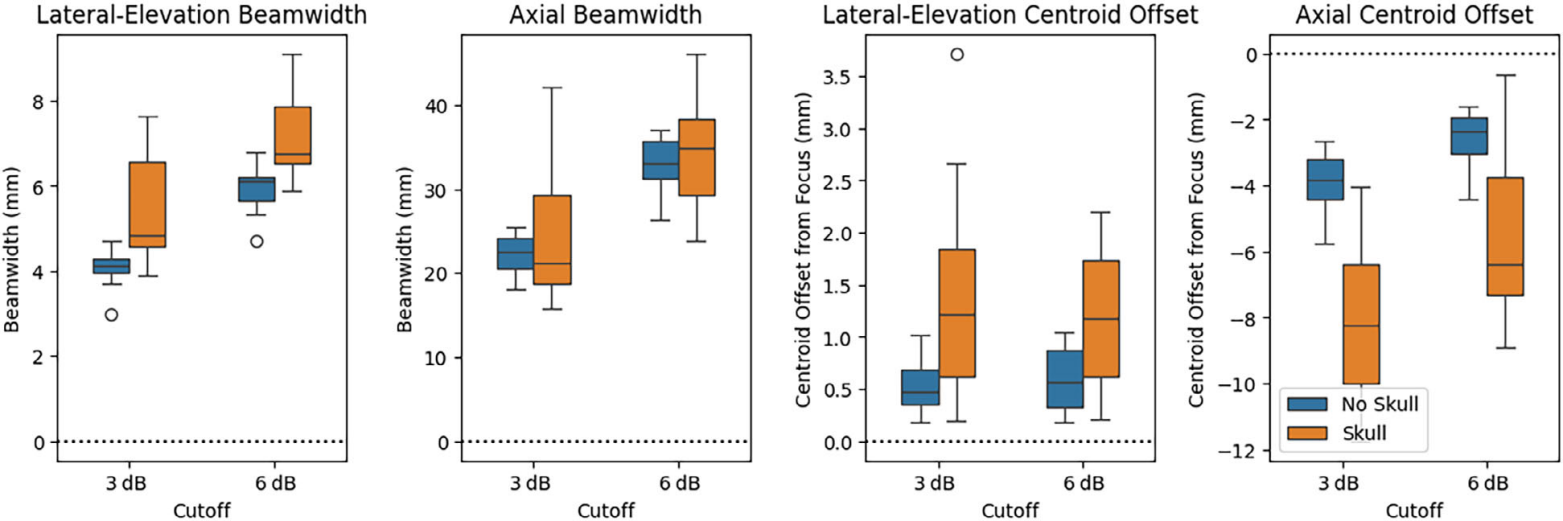
Position and size distortion of the focus due to the skull.

Table 7 shows the derated pressure value distributions across the three different target pressures. The subjects in Phase 1.2 had notably less favorable skull geometry compared with subjects in Phase 1.1 as after deration, the average pressure has not increased despite a higher “target” pressure setting. Figure 19 shows the effect of the skull on the lateral‐elevation and axial beamwidth, as well as the positional offset from the nominal focus, for both the centroid of pressures within the −3 and −6 dB contour cutoffs. The beam become marginally larger in the lateral‐elevation direction, but stays about the same size axially. The lateral‐elevation offset becomes larger (1.3 ± 0.9 mm for 3 dB) but is still substantially smaller than the original lateral‐elevation beamwidth (4.1 ± 0.4 mm). Similarly in the axial direction, the focus is offset by −8.1 ± 2.3 mm, which is smaller than the 22.2 ± 2.5 mm axial beamwidth.

**Table 7:** Derated Pressure Distribution Accounting for Skull Attenuation in the Three Phases

Target Pressure (MPa)	Count	Mean	Std	Min	Max
0.51	8	0.34	0.08	0.17	0.45
0.65	6	0.33	0.14	0.16	0.48
0.82	5	0.45	0.15	0.23	0.66

## Discussion

### System Capabilities

The system presented here offers unique advantages for precisely guiding LIFU to regions in the front of the brain. It combines a means of rapid positioning, using neuronavigation and a mechanical slider on the headset, with precise control of the targeting, through beamforming, allowing for quick probe placement without compromising accuracy. The probe's design allowed for a relatively coarsely‐diced and modestly‐sized matrix array, whose nominal steerable volume was ~3 × 3 × 3 cm, which encompassed the thermally‐safe steerable volume for the chosen intensities of ~1.8 × 2.5 × 2 cm. This design allowed the probe to reach targets up to 6 cm deep in most locations beneath the forehead. Compared with existing ultrasound neuromodulations technologies^21^ the system is wearable, does not require hospital equipment and is electronically steerable. While a fixed‐focus probe is simpler to operate and model, it has drawbacks. To reach the target, it needs precise positioning with a variable entry angle and distance from the head. This would require a bulky, external arm mechanism, making it unwearable. Our two‐pronged approach (physical and electronic steering) allows the array to be positioned in a way that avoids obstacles like sinuses, making it more versatile and maneuverable. And, although not used in this preliminary study, the system retains access to more advanced beamforming techniques, including element‐wise adjustment of apodizations and delays allowing for aberration correction. The small size of the array also means that the headset can be comfortably worn for the duration of the ultrasound without bulky fixturing, in contrast to current transcranial ultrasound arrays that require subjects to lay on a MRI machine throughout the treatment.^23^

### Volunteer Study

The study found successful placement of the headset and delivery of transcranial ultrasound in all participants. For all participants, there were no reported AEs or findings on the post‐delivery SWI images. Because the volunteers in this study were chosen to not have the type of abnormal neural connectivity associated with certain types of depression, it remains to be tested whether targeting the amPFC with this system will reduce depressive symptoms in those afflicted. Future work will also explore whether aberrant default‐mode network activity can be suppressed with tFUS in participants likely to have it, as well as assess the magnitude of such participants' clinical response to tFUS neuromodulation therapy.

### Post‐Study Research and Development

With observations made during the study about the presence of sinus occlusion along the ultrasound beam path, the sinus analysis and virtual‐fitting components were developed. Out of 19 participants analyzed, it was shown that only one had sinuses so large that it would not have been possible to reposition the probe to avoid blocking >30% of the elements, which would have improved dosing efficiency reliability across subjects. These methods will be incorporated into future studies. The segmented skull results helped quantify the loss of focal energy due to aberration in the skull and other tissue layers, and lay groundwork for aberration‐correction methods in the future, so that we might be able to provide additional compensatory energy than we did with the conservative approach used here, though careful consideration of the safety of any such correction methods will be necessary.

### Conclusions and Future Work

This neuromodulation system safety and useability study had LIFU delivered transcranially to the amPFC of 20 participants. This study assessed system usability, monitored AEs, and provided a neuroradiological readout of post‐delivery susceptibility‐weighted images that can probe for vascular micro‐hemorrhages. Although the dosing approach as conservative, there were no AEs or imaging findings, all participants tolerated the sessions well, and the users thought the interface was effective.

With the capabilities presented here and positive results of the feasibility/safety testing, this technology appears well‐suited to examine transcranial focused ultrasound's effect in the clinic. The next clinical work will explore the feasibility of the system to neuromodulate targets in participants with clinical depression, and future work will be needed to evaluate the dose–response curve.

## Data Availability

The data that support the findings of this study are available on request from the corresponding author. The data are not publicly available due to privacy or ethical restrictions.
